# Supplementary material for: TCF7L1 Controls the Differentiation of Tuft Cells in Mouse Small Intestine
Source: Cells. 2023 May 23;12(11):1452. doi: 10.3390/cells12111452 (PMC10253002; doi:10.3390/cells12111452)
Supplement: Supplementary file 1 [file cells-12-01452-s001.zip › Supplementary Figures.pdf]

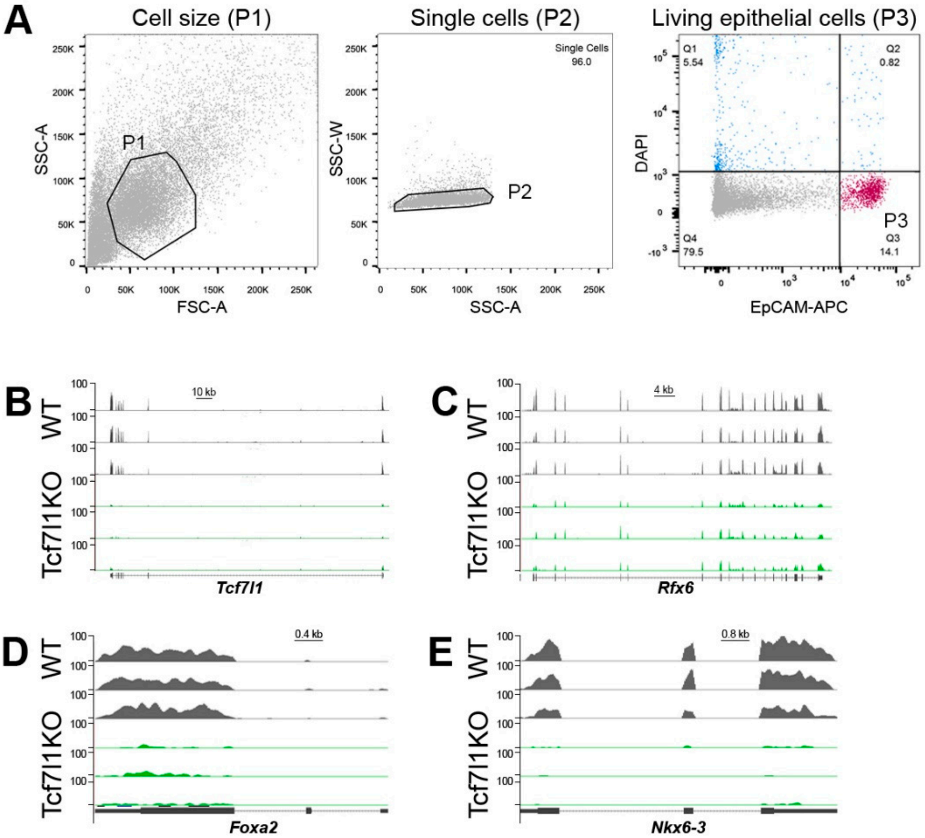

Figure S1. Transcriptional changes caused by loss of *Tcf7l1* in the embryonic gut epithelium (**A**) FACS strategy used to isolate EpCAM⁺ cells from the embryonic small intestine. Cells from the dissected small intestines at E13.5 were dissociated by enzymatic digestion and stained with EpCAM antibody. These cells were selected based on size (P1 and P2), DAPI dye exclusion and EpCAM⁺ labelling (P3) (n=3). (**B-E**) Gene expression patterns for *Tcf7l1* (B), *Rfx6* (C), *Foxa2* (D), and *Nkx6-3* (E) transcription factors in wild type (WT, grey) and $Shh^{Cre\text{-}EGFP}$:$Tcf7l1^{lox/lox}$ (Tcf7l1KO, green) embryonic intestinal epithelium at E13.5. The y-axis indicates the coverage normalized by library size (reads per million).

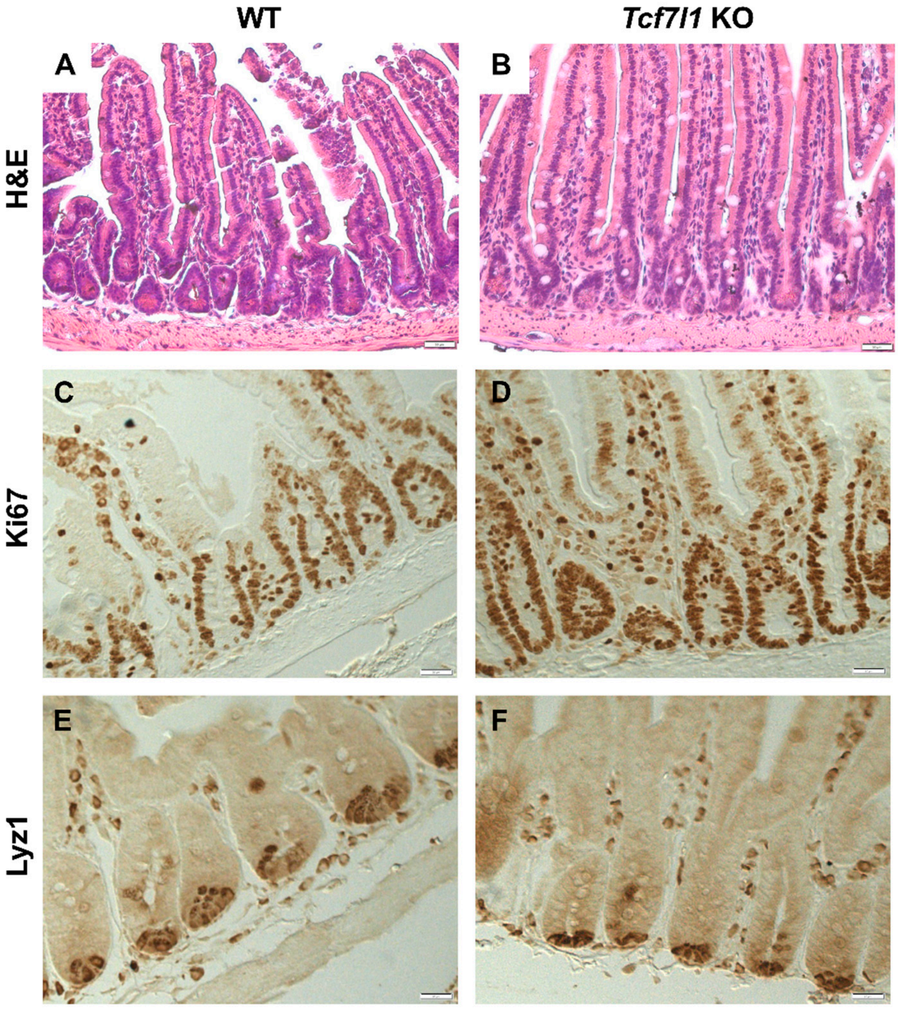

Figure S2. TCF7L1 is dispensable for the proliferation of transit-amplifying cell and the differentiation of goblet and Paneth cells. (**A-B**) Hematoxylin and eosin-stained section of the small intestine from adult wild-type (A) and *Shh*$^{Cre\text{-}EGFP}$:*Tcf7l1*$^{lox/lox}$ (B) mice. (**C-D**) Immunohistochemical detection of Ki67-positive cells showing proliferation in the adult small intestine from wild-type (C) and *Shh*$^{Cre\text{-}EGFP}$:*Tcf7l1*$^{lox/lox}$ (D) mice. (**E-F**) Lysozyme (LYZ1) immunohistochemistry in the adult small intestine of wild-type (E) and *Shh*$^{Cre\text{-}EGFP}$:*Tcf7l1*$^{lox/lox}$ (F) mice. Scale bar: 50µm (A-B) and 20 µm (C-F).
